# Responses of Cortical Neurons to Intracortical Microstimulation in Awake Primates

**DOI:** 10.1523/ENEURO.0336-22.2023

**Published:** 2023-04-25

**Authors:** Richy Yun, Jonathan H. Mishler, Steve I. Perlmutter, Rajesh P. N. Rao, Eberhard E. Fetz

**Affiliations:** Departments of 1 Bioengineering; 2 Physiology and Biophysics; 3 Allen School for Computer Science and Engineering; 4 Center for Neurotechnology; 5 Washington National Primate Research Center, University of Washington, Seattle, Washington 98195

**Keywords:** ICMS, neuronal excitability, nonhuman primate, short-term plasticity

## Abstract

Intracortical microstimulation (ICMS) is commonly used in many experimental and clinical paradigms; however, its effects on the activation of neurons are still not completely understood. To document the responses of cortical neurons in awake nonhuman primates to stimulation, we recorded single-unit activity while delivering single-pulse stimulation via Utah arrays implanted in primary motor cortex (M1) of three macaque monkeys. Stimuli between 5 and 50 μA delivered to single channels reliably evoked spikes in neurons recorded throughout the array with delays of up to 12 ms. ICMS pulses also induced a period of inhibition lasting up to 150 ms that typically followed the initial excitatory response. Higher current amplitudes led to a greater probability of evoking a spike and extended the duration of inhibition. The likelihood of evoking a spike in a neuron was dependent on the spontaneous firing rate as well as the delay between its most recent spike time and stimulus onset. Tonic repetitive stimulation between 2 and 20 Hz often modulated both the probability of evoking spikes and the duration of inhibition; high-frequency stimulation was more likely to change both responses. On a trial-by-trial basis, whether a stimulus evoked a spike did not affect the subsequent inhibitory response; however, their changes over time were often positively or negatively correlated. Our results document the complex dynamics of cortical neural responses to electrical stimulation that need to be considered when using ICMS for scientific and clinical applications.

## Significance Statement

Intracortical microstimulation (ICMS) is commonly used to probe the cortex, and previous studies have characterized the responses of single neurons to ICMS. However, these studies typically explored the averaged effects of ICMS throughout each experimental session, rather than by a trial-by-trial basis for each stimulation pulse. By shifting the approach, we explored the dependence of neural responses to ICMS on the spontaneous neural activity as well as the dynamics of responses over time produced by repetitive stimulation in awake nonhuman primates. Our results reveal how the responses of neurons to ICMS are related to interactions between local excitatory and inhibitory cortical circuits. These results will help inform the design of ICMS for both basic research and clinically relevant stimulation protocols.

## Introduction

Intracortical microstimulation (ICMS) is widely used for interfacing with the brain in both basic and clinical research, from inducing plasticity to employing sensory neuroprostheses in various animal models (Jackson and Fetz, 2011; Flesher et al., 2016; Hartmann et al., 2016; Lebedev and Nicolelis, 2017). The applicability of ICMS arises from the fact that it has the highest spatial and temporal specificity of all clinically applicable cortical stimulation techniques. However, the circuit mechanisms that drive the responses of neurons following ICMS, and the ways in which other factors such as timing and stimulation frequency affect the stimulus responses are not fully understood.

ICMS was originally thought to activate neural elements around the electrode tip. Regions closer to the tip would have higher activation in a sphere with an isotropic gradient, and the volume would grow with increasing current amplitude (Stoney et al., 1968; Ranck, 1975; Tehovnik et al., 2006). However, evidence shows that ICMS typically excites axons near the electrode tip that transsynaptically excite neurons up to several millimeters away (Gustafsson and Jankowska, 1976; McIntyre and Grill, 2000; Lesser et al., 2008; Histed et al., 2009; Logothetis et al., 2010; Hao et al., 2016). Additionally, the effects of ICMS are not limited to excitation, and includes a long-lasting inhibitory response that is commonly attributed to GABAergic synapses (Berman et al., 1991; Butovas and Schwarz, 2003; Hao et al., 2016).

Single neuron responses to ICMS are dynamic and can be modulated with repeated stimulation. The changes, in part, also depend on stimulus frequency. In particular, the excitation of neurons generally decreases over time and becomes more localized with higher frequencies (Dadarlat et al., 2019; Michelson et al., 2019). However, the reported frequency ranges and timescales are variable, and the driving processes remain unclear. The changes over time are often attributed to short-term synaptic plasticity or intrinsic plasticity of neurons which both depend on the frequency and pattern of stimulation (Zucker and Regehr, 2002; Abbott and Regehr, 2004; Citri and Malenka, 2008).

Altogether, these studies demonstrate that the effects of ICMS are not restricted to regions proximal to the electrode tip, and that responses consist of interplay between excitation and inhibition (Logothetis et al., 2010; Griffin et al., 2011; Borchers et al., 2012). Despite the ever-increasing understanding of how ICMS activates cortical circuits, several significant questions remain. How does the background neuronal activity, including firing rate and previous spike time impact the stimulus response? How do the responses change over time as a function of both the frequency of stimulation and proximity to stimulation site? Is the inhibitory response coupled to the excitatory response, or are they independently activated?

We addressed the questions above by delivering ICMS and examining responses of single neurons in primary motor cortex (M1) of three macaque monkeys with chronically implanted Utah arrays. Single-pulse ICMS was delivered to one channel for up to 20 min while the spikes of single neurons were simultaneously recorded from all other electrode channels. We tracked the probability of evoking spikes as well as the duration of the evoked inhibition and varied both the sites and frequency of the stimulation between sessions. Our results expand on previous findings by characterizing the dependencies of the neuronal responses to background neuronal activity, distance, and stimulation frequency, and exploring the interactions between the excitatory and inhibitory responses.

## Materials and Methods

### Experimental design

#### Implants and surgery

Three male pigtail macaque monkeys (*Macaca nemestrina*) were unilaterally (right hemisphere) or bilaterally implanted with 96-channel Utah microelectrode arrays (Blackrock Microsystems; 10 × 10, 400-μm interelectrode distance, 1.5-mm depth, Iridium oxide) in the hand region of M1. Sterile surgeries were performed under isoflurane anesthesia and aseptic conditions with continuous monitoring of all vitals. Animals received postoperative courses of analgesics and antibiotics following each surgery. All procedures conformed to the National Institutes of Health *Guide for the Care and Use of Laboratory Animals* and were approved by the University of Washington’s Institutional Animal Care and Use Committee.

Implantation of the arrays was guided via stereotaxic coordinates. A 1.5-cm-wide square craniotomy centered at 4 mm lateral of bregma was performed to expose the dura. Three sides of the exposed dura were cut to expose the cortex, after which a Utah microelectrode array was implanted. Two reference wires were inserted under the dura and two were inserted between the dura and the skull. The dura was then sutured around the implant, and the bone flap from the craniotomy was reattached to the skull with a titanium strap and titanium skull screws. A second, smaller titanium strap was screwed onto the skull to secure the wire bundle that connected the array to a connector “pedestal” that was also secured to the skull with eight skull screws. The skin incision was then sutured around the pedestal base.

To facilitate the chronic recording of neuronal activity, the monkeys were also implanted with halos made with 3/8” aluminum bars in an egg-shaped oval that was 17 cm long and 15.3 cm wide. Four titanium straps were affixed to the skull via titanium bone screws. Two of the straps were implanted bilaterally over the occipital ridge, and two were bilaterally implanted temporally. After the plates integrated with the skull for six weeks, the halo was secured to the skull with four pins, each of which were seated in one of the four plates.

#### Electrophysiology

Stimulation and recording of single-unit activity were conducted with one of three systems: (1) Neurochip3 [custom bidirectional brain-computer interface developed in our laboratory (Shupe et al., 2021), 32 channels, 20-kHz sampling rate], (2) Neural Interface Processor (Ripple Neuro, 96 channels, 30 kHz sampling rate), or (3) RZ2 BioAmp Processor, PZ5 NeuroDigitizer Amplifier, and IZ2 Electrical Stimulator (Tucker-Davis Technologies, 96 channels, 25-kHz sampling rate).

#### Experimental setup

**Figure 1.**
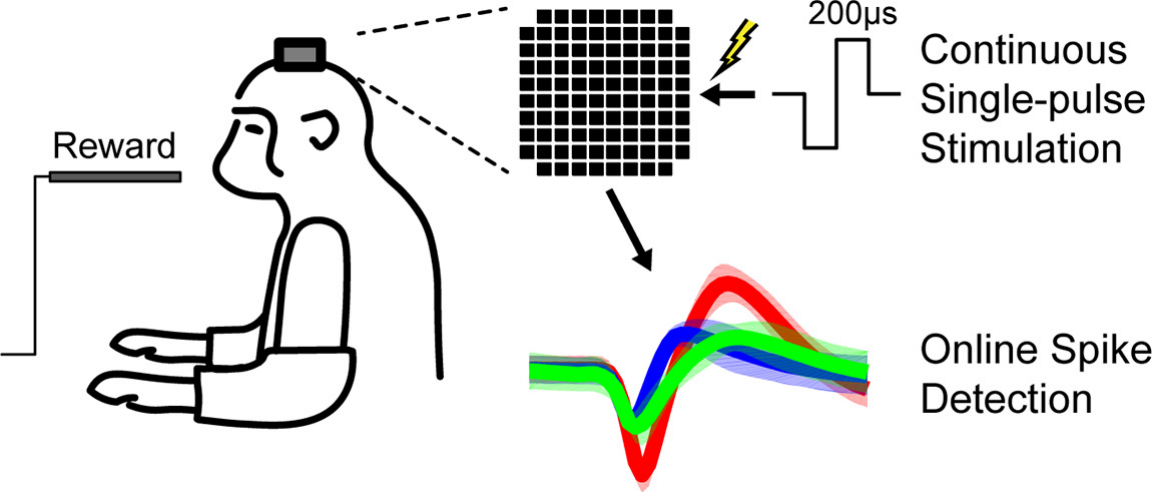
Experimental setup and timeline. Macaques calmly sat in a chair receiving apple smoothie reward through the experiment. Cathodic, 200-μs phase width, single-pulse ICMS was delivered to one channel of the Utah array in primary motor cortex while unit responses were recorded across the array. Each session consisted of a prestimulus and stimulus epoch.

The monkeys were trained to calmly sit in a primate chair while periodically receiving an apple smoothie reward without performing a task (Fig. 1). Each session included a prestimulus epoch lasting between 5 and 10 min and a stimulus epoch lasting between 5 and 20 min. During the stimulus epoch we delivered tonic single pulse stimulation (cathodal, biphasic, 200-μs phase width) to a single channel at rates between 1 and 20 Hz. For testing the effects of current amplitude, a range of 2–50 μA was used. Current amplitude was fixed at 15 μA for all other experiments and analyses. The stimulation frequency was fixed during the stimulus epoch for each session.

Experiments with tonic stimulation were unable to be used to measure the evoked spike probability of the units as a function of the time delay between their previous spike times and stimulation onsets because of a low sampling density of various time delays between 0 and 50 ms. For this analysis, we performed a separate set of experiments involving 18 distinct units in which we delivered Poisson distributed single pulse stimulation (cathodal, biphasic, 15 μA, 2000-μs phase width) to a single, or multiple channels at rates between 2 and 12 Hz.

### Data analysis

#### Evoked spike acquisition

**Figure 2.**
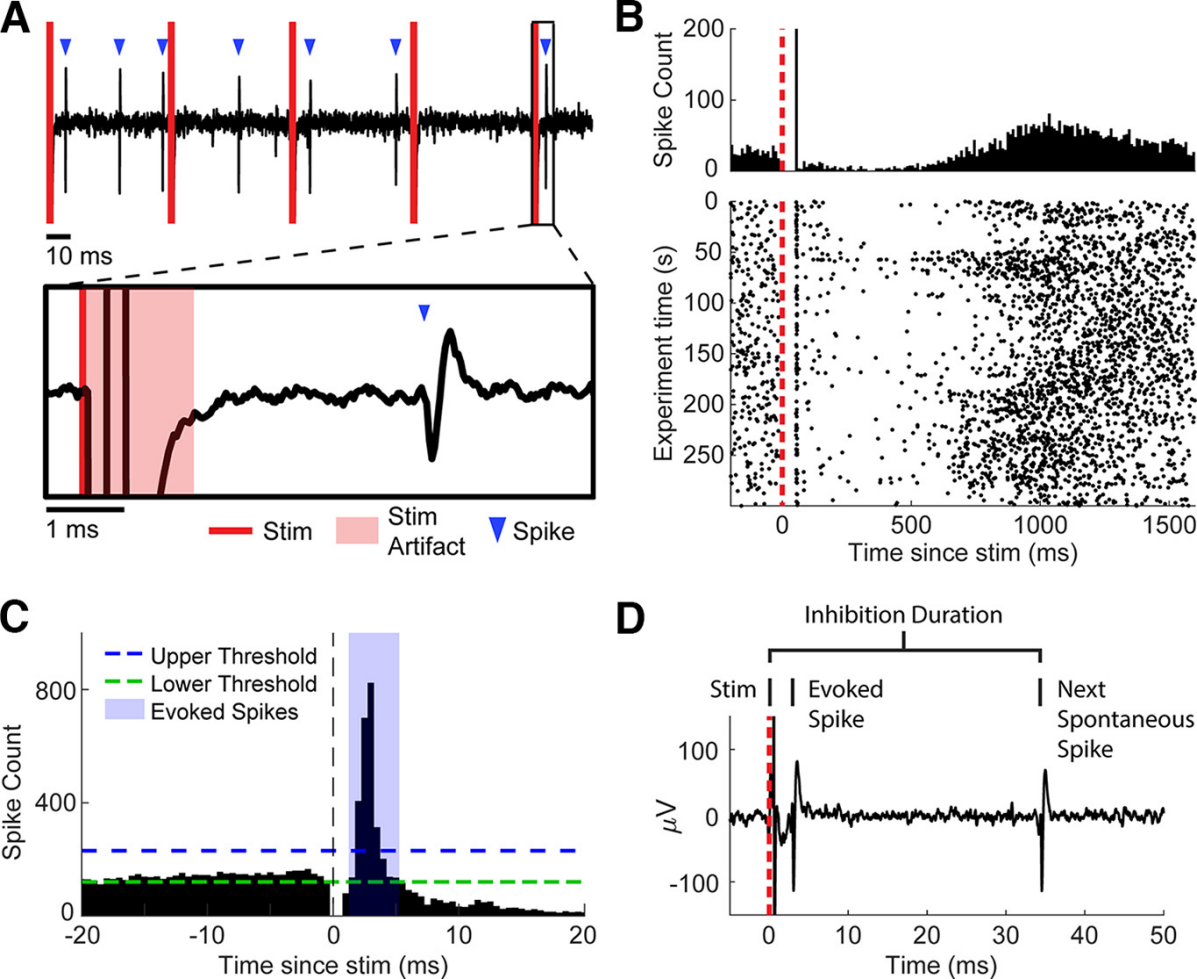
Detection of evoked spikes and inhibition. ***A***, Example of filtered data trace. The inset shows a stimulus followed by an evoked spike after 3.5 ms. ***B***, Example PSTH (top) and corresponding raster plot (bottom). ***C***, Defining evoked spikes. A PSTH with 0.5-ms bins was generated. Peaks after the time of stimulation greater than the upper threshold (mean + 2 SDs of −20 to −2 ms in the PSTH) down to the lower threshold (mean − 2 SDs) were called evoked spikes. ***D***, Defining inhibition. Rather than using the PSTH, the inhibition duration was calculated for each stimulus by taking the time from stimulus onset to the next spontaneous spike. Stimulus intensities were 15 μA, unless stated otherwise.

Spikes were sorted using two time-amplitude windows, initially online and subsequently confirmed offline. Stimulus artifacts lasted around 1.1–1.6 ms. Spikes were frequently detected immediately following the artifact (Fig. 2*A*). The timing of evoked spikes was found by calculating the peristimulus time histogram (PSTH; 0.5-ms bin widths) of spikes in the window from −20 to 20 ms from the time of stimulation (Fig. 2*B*). To isolate the evoked spikes from the spontaneous activity, we defined upper and lower thresholds in the PSTH as the histogram mean plus or minus two times the SD from −20 to −2 ms. We then found the largest peak in the PSTH from 1 to 15 ms after stimulation that was larger than the upper threshold and tracked adjacent bins in both directions until we reached the lower threshold on both sides. All spikes occurring within this window were denoted as stimulus-evoked spikes (Fig. 2*C*). If no peak was greater than the threshold the spike was not considered to have been evoked by stimulation. The probability of evoked spikes was calculated as the number of evoked spikes divided by the number of total stimuli. For any analysis over time, the evoked spike probability was calculated for stimuli within overlapping 30-s bins with 1-s steps.

We tracked a total of 148 distinct units across all experiments with tonic stimulation, and 18 for those with Poisson stimulation. Experiments characterizing the base stimulus responses used unique pairs of stimulated site and recorded spike (*n* = 420). Experiments assessing changes over time used unique trios of stimulated site, stimulation frequency, and recorded spike (*n* = 585).

#### Inhibitory response acquisition

**Figure 3.**
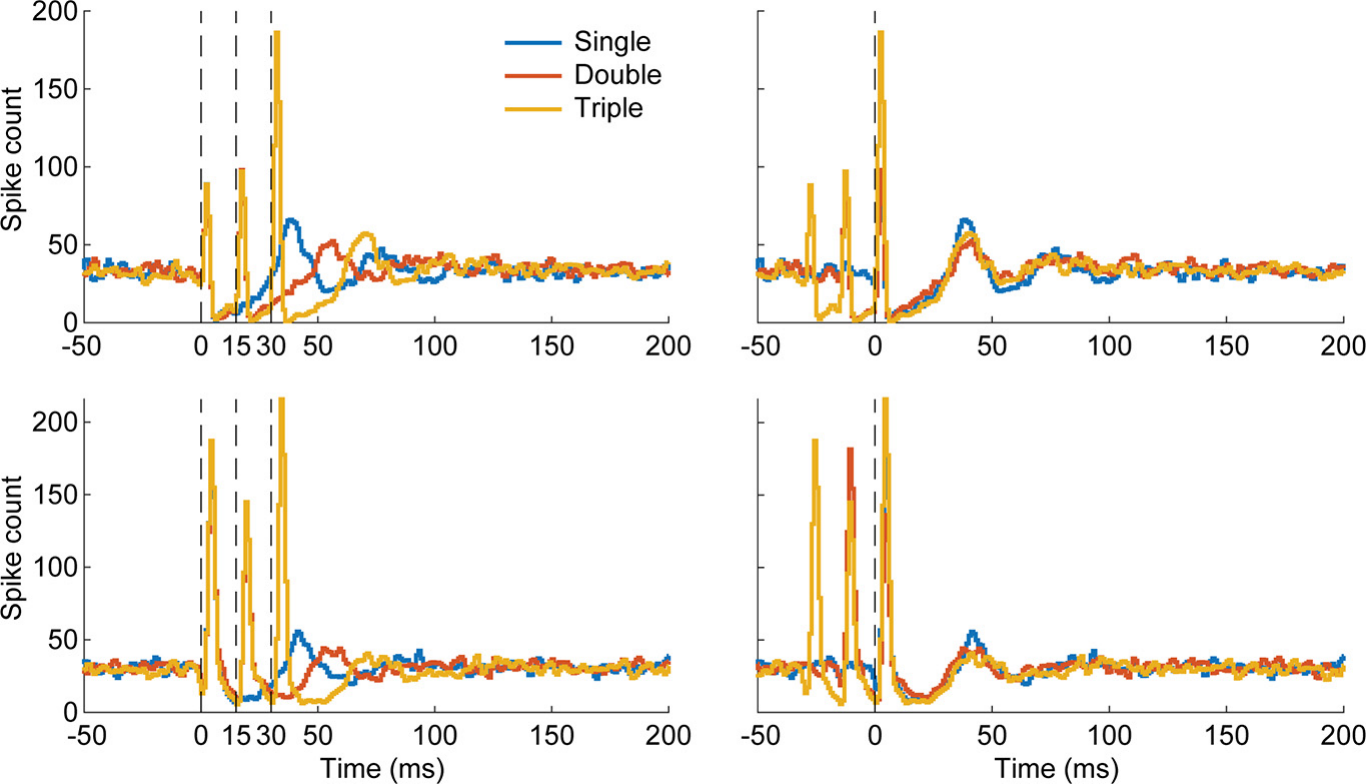
Stimulation during inhibition. Two representative examples of double-pulse and triple-pulse stimulation in which subsequent pulses arrive during the inhibitory response of the previous pulse (left). Spikes were readily evoked even when stimulating during the inhibitory response. Aligning the PSTHs to the final stimulus pulse (right) shows that the inhibition restarts at each stimulus pulse. Each condition consisted of 1500 stimuli. Bin width: 1 ms.

The inhibitory response was measured using the PSTH in previous studies (Butovas and Schwarz, 2003; Hao et al., 2016). While evoked spike timing can easily be determined with the PSTH because of their high probability and narrow time window, the duration of the inhibitory response occurs over a broader window of time. Therefore, a large number of stimuli is required to get a densely sampled PSTH to measure inhibition strength, which prevents measuring changes in inhibitory strength with fine temporal resolution. Thus, rather than using the PSTH, we measured the duration of inhibition on a stimulus-by-stimulus basis by calculating the time between the onset of each stimulus and the first nonevoked spontaneous spike (Fig. 2*D*). If a second stimulus occurred before a spontaneous spike, the duration of inhibition was measured with respect to the second stimulus rather than the first, as we found that each stimulus “reset” the period of inhibition (Fig. 3). For any analysis over time, we calculated the median inhibition duration of stimuli within overlapping 30-s bins with 1-s steps.

#### Single-unit response dependencies

Changes in evoked spike probability and inhibition duration because of changes in stimulation amplitude were fitted to a sigmoidal curve given by:

(1) $$y=\frac{1}{a_{1}+e^{-(x-a_{2})/a_{3}}}+a_{4},$$

where x is the stimulus amplitude, y is the evoked spike probabilities or inhibition duration, and $a_{1,2,3,4}$ are fitted variables.

To calculate the spontaneous firing rate for each unit over time, we disregarded the times from each stimulus onset to the next spontaneous spike. This effectively removed the stimulus response from the firing rate calculation, providing us with an independent measure of spontaneous activity.

The autocorrelation histograms for the 18 units in which we characterized the evoked spike probability as a function of the time delay between their previous spontaneous spike times and stimulation onsets were calculated from their respective prestimulus epochs. This analysis was also repeated for time delays between the units’ previous evoked spike times and stimulation onset. The histograms were binned between 0 and 50 ms in nonoverlapping 1-ms bins. For both the spontaneous and evoked analyses, we excluded any unit that did not have at least five samples in at least 70% of the bins.

The dependencies of evoked spike probabilities on the timing of stimulation were calculated by first measuring the delays between each stimulus and the preceding spike. We separated this into two groups corresponding to whether the preceding spike was spontaneous or evoked. In some instances, a spike, be it evoked or spontaneous, was followed by two stimuli with no spike in between. We discarded the second stimulus of all such stimuli from these analyses to remove any confounds from multiple stimuli on the probability of evoking a spike.

For each unit, there were unique, non-zero transmission delays between the onset of stimulation and when the evoked excitatory signal arrived at the unit to evoke it. Since each unit had a different average evoked spike latency, adding the transmission delays to the stimulation delays served as a means of normalizing the effect. Therefore, when we calculated the probability of evoking a spike as a function of the delay between the previous spike time and stimulation onset, we modified the delays by adding the average evoked spike latency of the unit to each of the delays. We then calculated the probability of evoking a spike for stimuli with delays from 0 to 50 ms with 1-ms time bins, then applied a moving average with a 5-ms window. Since the average spike latencies were added to each delay, time bins that were less than the average evoked spike latency were not included in correlation calculations.

#### Changes over time

Pairwise correlations and their statistical significance between firing rate, evoked spike probability, and inhibition duration over time were calculated using the Pearson correlation coefficient r:

(2) $$r=\frac{\sum^{}(x_{i}-\bar{x})(y_{i}-\bar{y})}{\sqrt{\sum^{}{\left(x_{i}-\bar{x}\right)}^{2}\sum^{}{\left(y_{i}-\bar{y}\right)}^{2}}},$$

where $\bar{x}$ is the mean of the *x*-variable, and $\bar{y}$ is the mean of the *y*-variable. Firing rate was calculated by removing all times between each stimulus onset to the first spontaneous spike (the inhibitory period) to remove any confounding affects between firing rate and the inhibitory response.

Linear and exponential fits were performed on binned evoked spike probabilities and inhibition durations to determine changes over time because of repetitive stimulation:

(3) Linear y=a * t + b

(4) $$\mathrm{Exponential}\;y=a+b*{\left(1-c\right)}^{t},$$

where y is either the evoked spike probability or inhibition duration, t is time and a, b, and c are the fitted variables. Changes were denoted to occur if the ANOVA *F*-statistic resulted in *p* < 0.05. The sign of the linear fit slope ( a in Eq. 3) or the sign of the exponent base ( b in Eq. 4) of the exponential fit determined whether the changes were classified as increasing or decreasing. Unit-stimulation site pairs with less than 3% average probability of evoking spikes were disregarded for analyses over time because of their inconsistency. The changes over time in spikes and inhibition were designated to be correlated if their correlation had a *p*-value less than 0.05.

### Statistical analysis

Two-sided Wilcoxon signed-rank test (*signrank*, MATLAB) was used to compare between groups because of the nonparametric nature of the data. Two-sided Wilcoxon rank-sum (*ranksum*, MATLAB) tests were used for paired data. Fisher’s exact test (*fishertest*, MATLAB) or two-way ANOVA (*anova2*, MATLAB) was used to compare pairs of or multiple groups of categorical data, respectively. The Pearson correlation was used for all correlation tests. The Kolmogorov–Smirnov test was used as a nonparametric test to compare probability distributions; *p*-values for significance and tests used are reported in individual analyses.

## Results

### Evoked spikes and inhibitory response

We found that ICMS in an awake animal sitting calmly elicited a brief excitatory response in units followed by a longer period of inhibition. Electrodes on the Utah array typically showed evoked spikes occurring 1.5–10 ms after single-pulse stimulation. The inhibition typically followed the excitatory response and was observed as the suppression of firing in the PSTH for 5–100 ms, although in some instances it lasted up to 200 ms. We also observed that stimulus-evoked inhibition could occur in the absence of the excitatory response.

To ensure that stimulation arriving during inhibition was not affecting the stimulus response, we delivered trains of two or three pulses with each subsequent pulse timed to occur during the inhibitory response of the previous stimulus. Single, double, and triple pulse stimuli were delivered to a single channel, randomly interleaved at 2 Hz. Our results across five different sessions show that stimuli delivered during the inhibitory response were able to reliably evoke spikes comparable to when stimuli was delivered at other times, as previously reported (Butovas and Schwarz, 2003; Fig. 3, left). Furthermore, each stimulus pulse “reset” the inhibitory response such that the duration of inhibition was the same following each pulse train (Fig. 3, right).

### Effects of distance from stimulation site and stimulus amplitude

**Figure 4.**
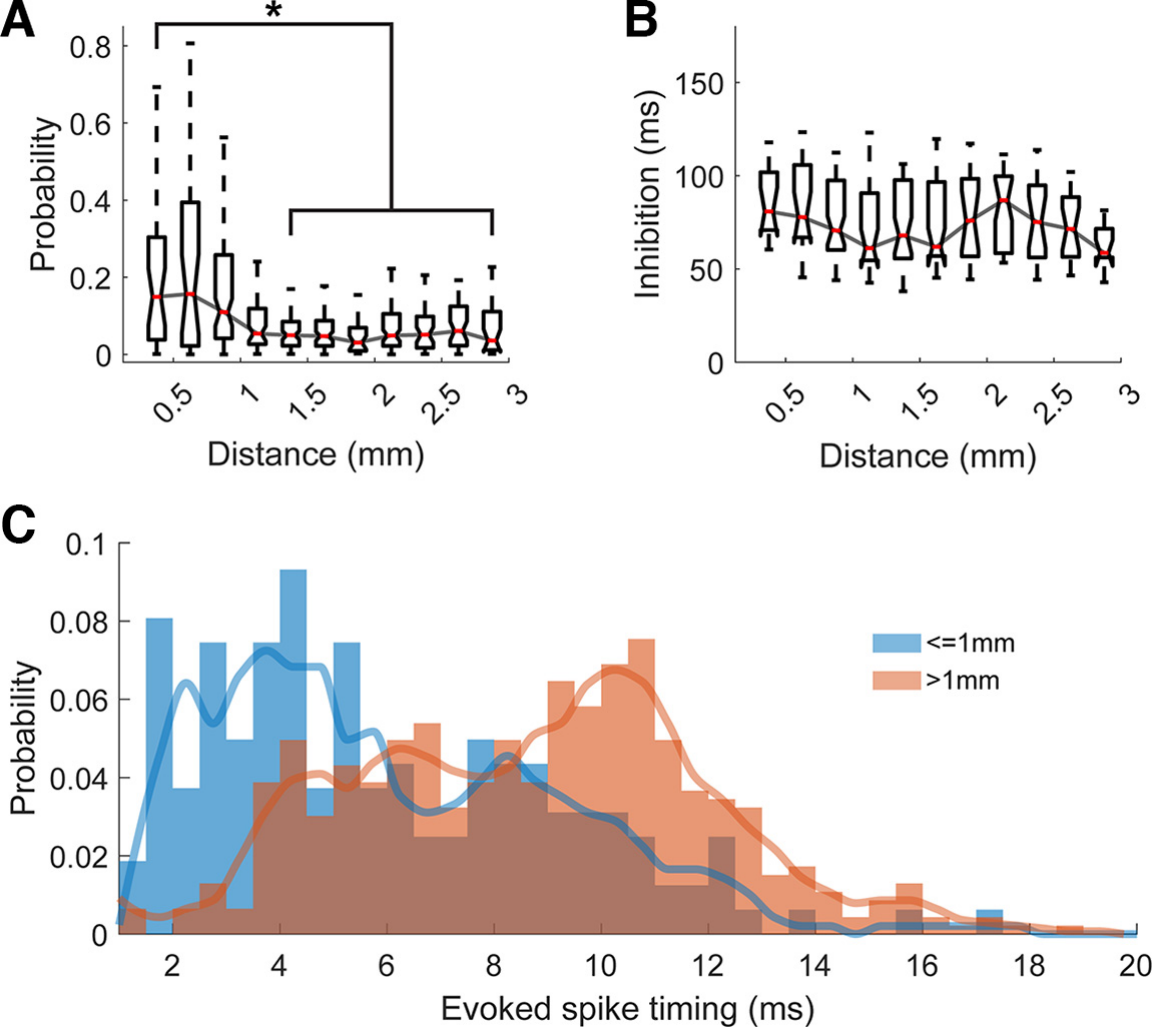
Effect of distance from stimulus site. ***A***, Evoked spike probability with respect to distance from the stimulated site in 0.25-mm bins. Stars (*) denote statistical significance from the closest group (ANOVA, *p* < 0.05). ***B***, inhibition duration with respect to distance from the stimulated site in 0.25-mm bins. No groups were statistically different from the closest group (ANOVA). ***C***, Probability histogram of evoked spike timings split into sites close (<1 mm, *n* = 121) to the stimulated site and all other sites (*n* = 299). The line shows the cubic interpolated moving average over three bins.

Evoked spikes occurred with greater probability and less variable latencies for units close to the stimulus electrode than for more distant units. The probability of evoking a spike in units <1 mm from the stimulus site was significantly greater than for further sites (Fig. 4*A*). In addition, units in closer sites on average had evoked spikes that occurred at shorter latencies (Wilcoxon rank sum test, *p* = 8.5e-29), suggesting the presence of monosynaptic and polysynaptic activation (Fig. 4*C*). The duration of inhibition did not have this trend: recording sites <1 mm from the stimulus site did not have significantly different duration of inhibition compared with sites farther away (Fig. 4*B*).

**Figure 5.**
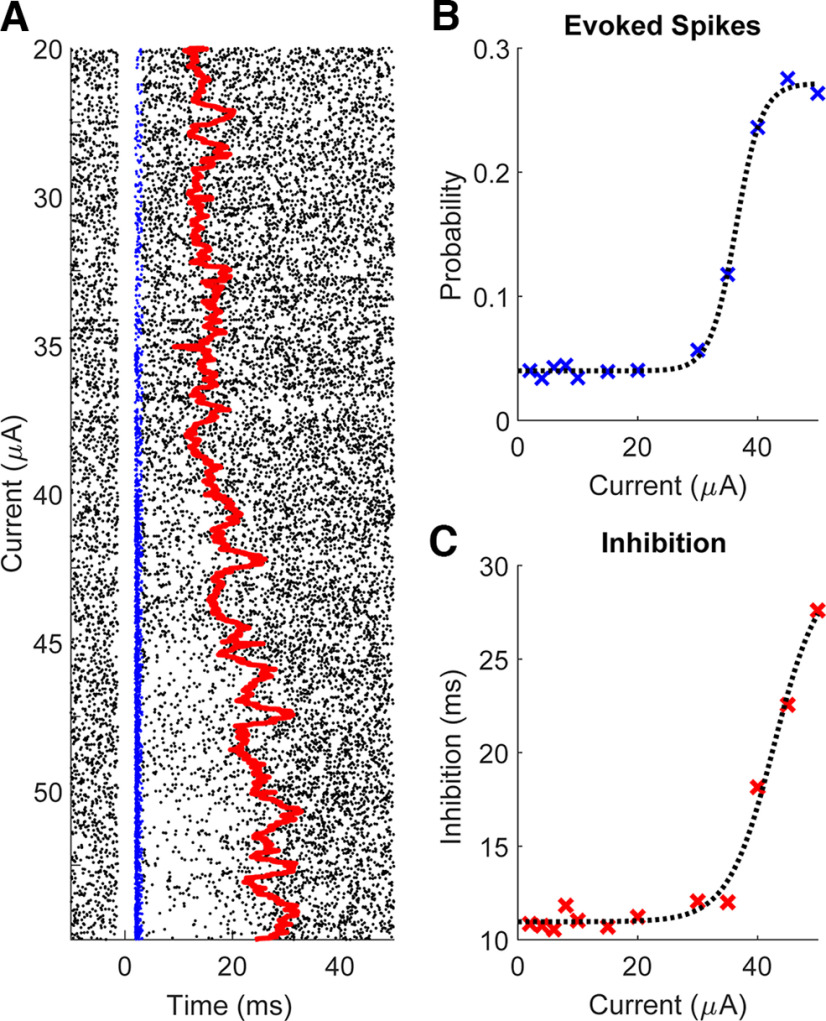
Effect of stimulus amplitude. ***A***, An example raster plot of a unit over different stimulus current amplitudes delivered for 5 min each at 2 Hz. Blue dots represent evoked spikes and the red line shows the median of the inhibition duration binned every 30 s with 1-s steps. ***B***, An example evoked spike probability as a function of amplitude and (***C***) inhibition duration as a function of stimulus amplitude. The dashed lines are fitted sigmoidal curves using least-squares regression.

In contrast, both the probability of evoking spikes and the duration of inhibition increased sigmoidally with the stimulus amplitude for all responsive units (Fig. 5). The sigmoid curves were always steep: a change of 10–20 μA in stimulus intensity generated the difference between 5% and 95% of the maximum value for both evoked spike probability and inhibition duration.

### Stimulus responses are not driven by an underlying state

**Figure 6.**
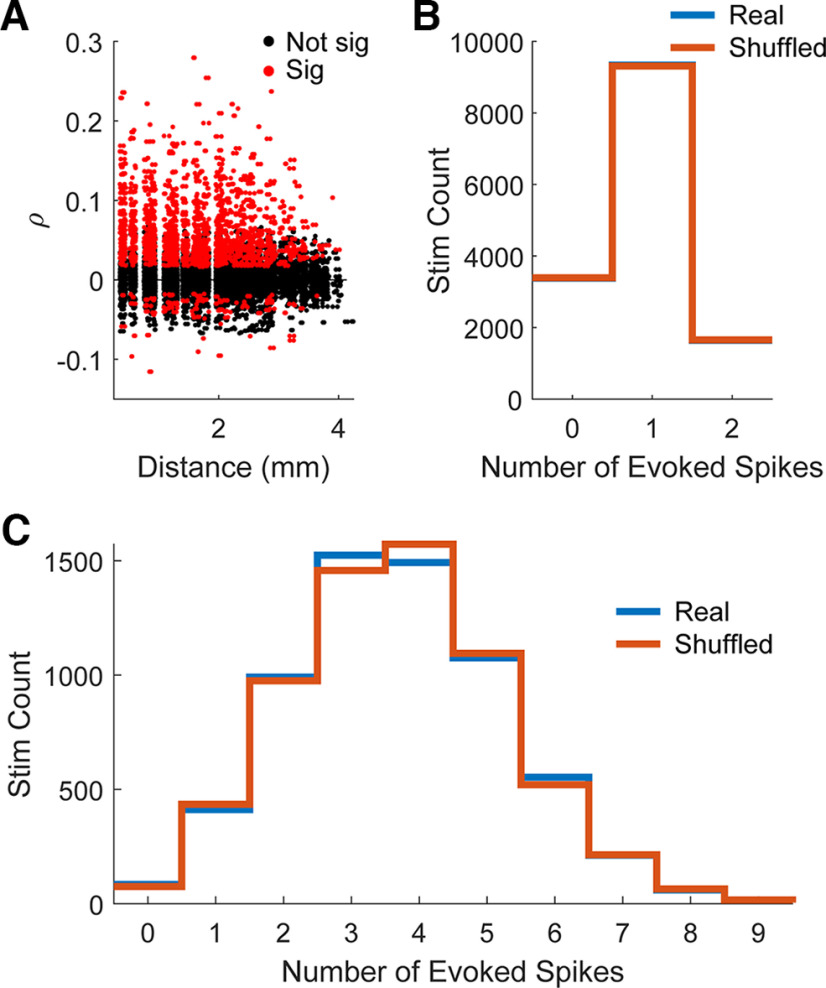
Evoked spikes are not driven by an underlying state. ***A***, Scatter plot showing the correlation coefficients of evoked spike activation for pairs of units with respect to distance between the recorded channels (*n* = 12,419 pairs). Red points show pairs of units that were significantly likely to be co-activated. Units that were farther away from each other were less likely to be co-activated. (Sig: statistically significant, *p* < 0.05; Not sig: not statistically significant). ***B***, An example of distributions of stimuli that evoked a specific number of evoked spikes using the true data (Real) and when the evoked spikes were shuffled (Shuffled) for a pair of spikes. The two distributions are not statistically different (Kolmogorov–Smirnov test, *p* > 0.05). ***C***, An example of distributions similar to ***B*** but extended to all evoked spikes within a single session. The two distributions are not statistically different (Kolmogorov–Smirnov test, *p* > 0.05).

Stimulus-evoked spikes are likely mediated through the activation of cortical circuitry, the stimulation excites a group of fibers near the electrode tip which projects onto the neurons being recorded (Gustafsson and Jankowska, 1976; Butovas and Schwarz, 2003; Histed et al., 2009; Logothetis et al., 2010). Subsequently, there may be covarying responses between different spikes because of activation via similar cortical paths. Thus, we determined whether pairs of units were likely to have the same responses for individual stimuli. Figure 6*A* shows the Pearson correlation coefficient between pairs of evoked spikes plotted against distance from one another.

Although there exist statistically significant correlations (2904 out of 12,419 tested pairs, or ∼23%), this may be a consequence of the units within the pairs independently having high probabilities of being evoked, rather than being correlated because of an underlying network state, thereby artificially inflating their correlation strength. To test for this, for each unit in each pair, we first counted the number of evoked spikes that occurred over the experiment, and tracked which stimuli evoked them. We then randomly shuffled which stimuli evoked each of the spikes, thereby eliminating any stimulus-by-stimulus relationship in the evoked activity of the pairs. We then compared the two histograms, one for true and one for the shuffled data, of the number of stimuli that evoked spikes in 0, 1, or both units (example shown in Fig. 6*B*). If there truly is a relationship between pairs of spikes, the histograms should be different from one another. The procedure was repeated 100 times to ensure the shuffling properly captured the baseline. We found that on average 1013 (±8.3 SD) out of the 12,419 tested pairs (∼8.2 ± 0.06% SD) had significantly different distributions (Fisher’s exact test, *p* < 0.05), but a large majority did not.

Although the relationship between pairs of units was weak, there may be a population wide brain state that is driving the responses. We performed similar analysis as before but across an entire session, for each unit in each session we randomly shuffled which stimulus evoked a spike to eliminate any causal relationship between the network state and responses to stimulation. We found that the real and shuffled histograms of the number of spikes evoked by each stimulus were not significantly different from one another in any session throughout all repetitions. A representative example of the distributions is shown in Figure 6*C*.

### Spontaneous activity affects stimulus responses

**Figure 7.**
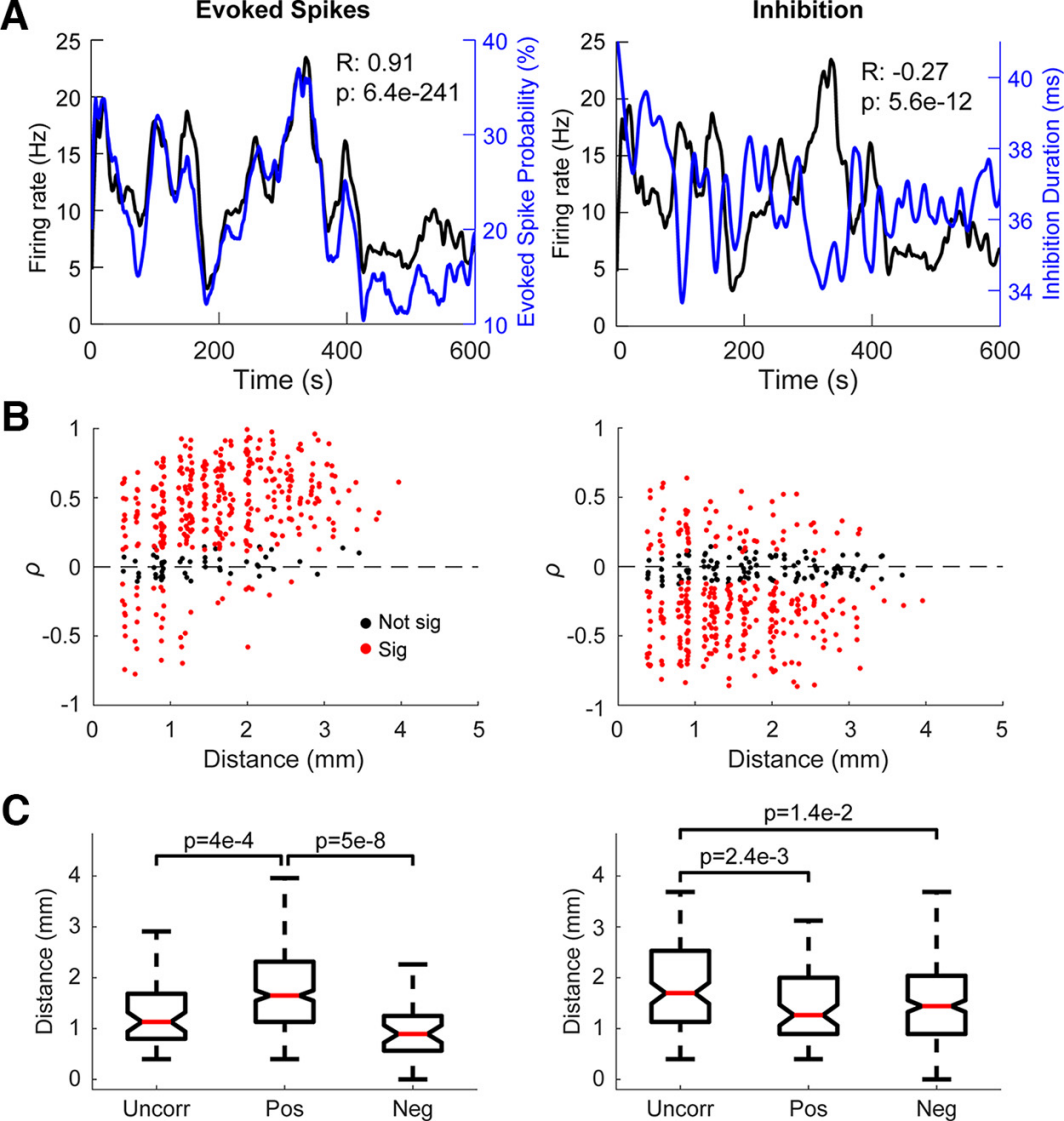
Probability of evoking a spike and inhibition duration are related to spontaneous firing rate. ***A***, An example of a neuron with positively correlated firing rate (black) and evoked spike probability (blue) over 10 min (left), and a neuron with a negatively correlated firing rate (black) with inhibition duration (blue) over 10 min (right). The rate and probabilities are averaged over 30-s bins with 1-s steps. ***B***, Scatter plot of the Pearson correlation coefficient (*ρ*) between the spontaneous firing rate and the probability of evoking spikes (left) or the inhibition duration (right) against the distance of the recorded unit from the stimulated site (*n* = 420). ***C***, Distance from the stimulated site for units with uncorrelated, positively correlated, and negatively correlated evoked spike probabilities (left) or inhibition duration (right) with firing rate. Labeled *p*-values are from the Wilcoxon rank-sum test.

In addition to stimulation current and separation of the recording and stimulation sites, we found two other dependencies that affect the response. One is the spontaneous firing rate of the recorded units. Both the evoked spike probability and inhibition duration had statistically significant correlations with spontaneous firing rate over time (Fig. 7). Table 1 documents the number of units with uncorrelated, positively correlated, or negatively correlated evoked spike probability and inhibition duration with spontaneous firing rate. A slight majority of units tested (300/585, 51%) had evoked spike probabilities that were positively correlated with firing rate and inhibition duration that were negatively correlated with firing rate. We additionally performed a two-way ANOVA to determine whether the two relationships were dependent on one another but found no significant relationship (*p* = 0.11). Units with positively correlated evoked spike probabilities and spontaneous firing rate were typically farther from the stimulated site than units with a negatively correlated relationship (Fig. 7*B*, left, *C*, left). In contrast, units with positively correlated inhibition duration and spontaneous firing rate were typically closer to the stimulated site (Fig. 7*B*, right, *C*, right).

**Table 1:** Evoked spike probability and inhibition duration correlations with spontaneous firing rate

		Inhibition duration and spontaneous firing rate			
		Uncorrelated	Positive correlation	Negative correlation	Total
Evoked spike probability and spontaneous firing rate	Uncorrelated	10	18	37	65
	Positive Correlation	86	77	300	463
	Negative Correlation	12	13	32	57
	Total	108	108	369	585

**Figure 8.**
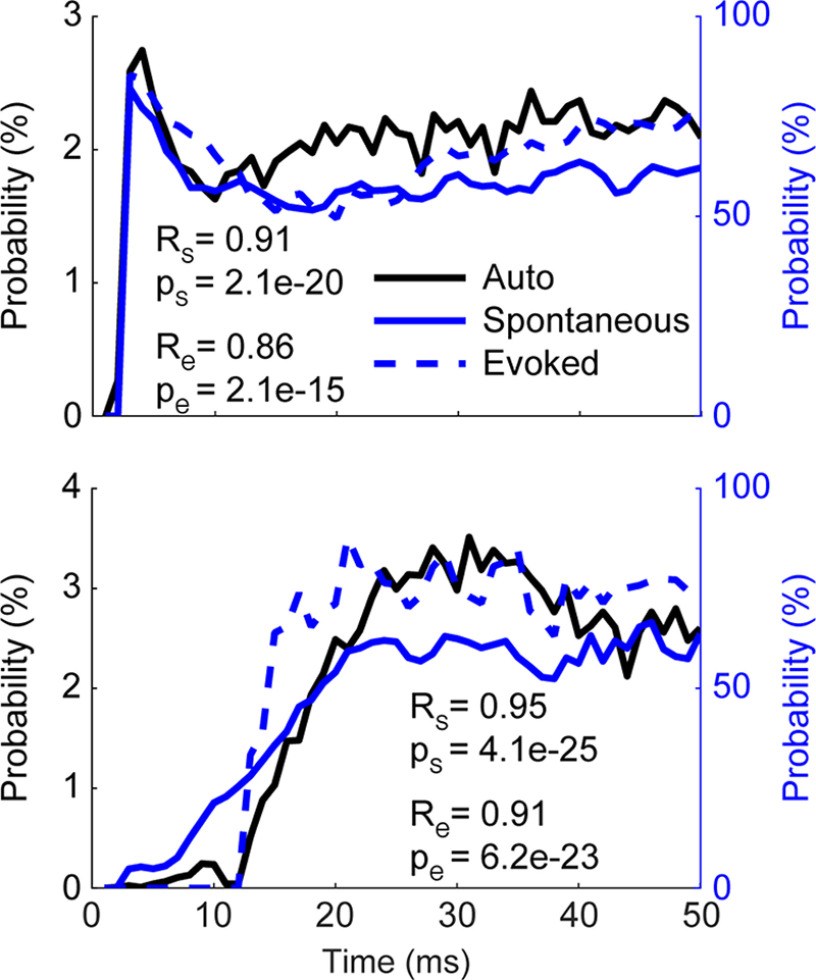
Probability of evoking a spike depends on the timing of stimulus. Three examples of unit autocorrelations (Auto Cor), and probability of a stimulus evoking a spike relative to timing from the most recent spontaneous (Spont) and evoked (Evoked) spike. The plots show two different autocorrelation waveforms with correlated evoked spike probability. All traces show a moving average using 5-ms bins with a 1-ms step size. R_s_ is the correlation coefficient between Auto Cor and Spont, R_e_ the correlation coefficient between Auto Cor and Evoked, and p_s_ and p_e_ are the corresponding *p*-values of correlation.

The second dependency was the timing of stimuli relative to the most recent spike. We analyzed 18 separate units (three from monkey S, four from monkey K, and 11 from monkey J). In 16 of the 18 units (three from monkey S, four from monkey K, and nine from monkey J), the probability of evoking a spike varied as a function of the time between the onset of stimulation and the most recent spontaneous spike. For these 16 units, the probability was significantly positively correlated with the unit’s autocorrelogram in the absence of stimulation (Fig. 8). In the four units that met the inclusion criteria (one from monkey S, three from monkey K), their evoked spike probability distributions were the same even if the most recent spike was evoked, rather than spontaneous. The inhibitory response did not depend on the timing of prestimulus spikes.

In one of the two units without a positive correlation, the probability of evoking a spike did not depend on the timing between the previous spike and stimulus onset, while the other had a negative correlation. However, in both of these units, the probability of evoking a spike was consistently high, which suggests that the stimulation amplitude was large enough to evoke spikes regardless of other properties.

### Repetitive stimulation changes stimulus response over time

**Figure 9.**
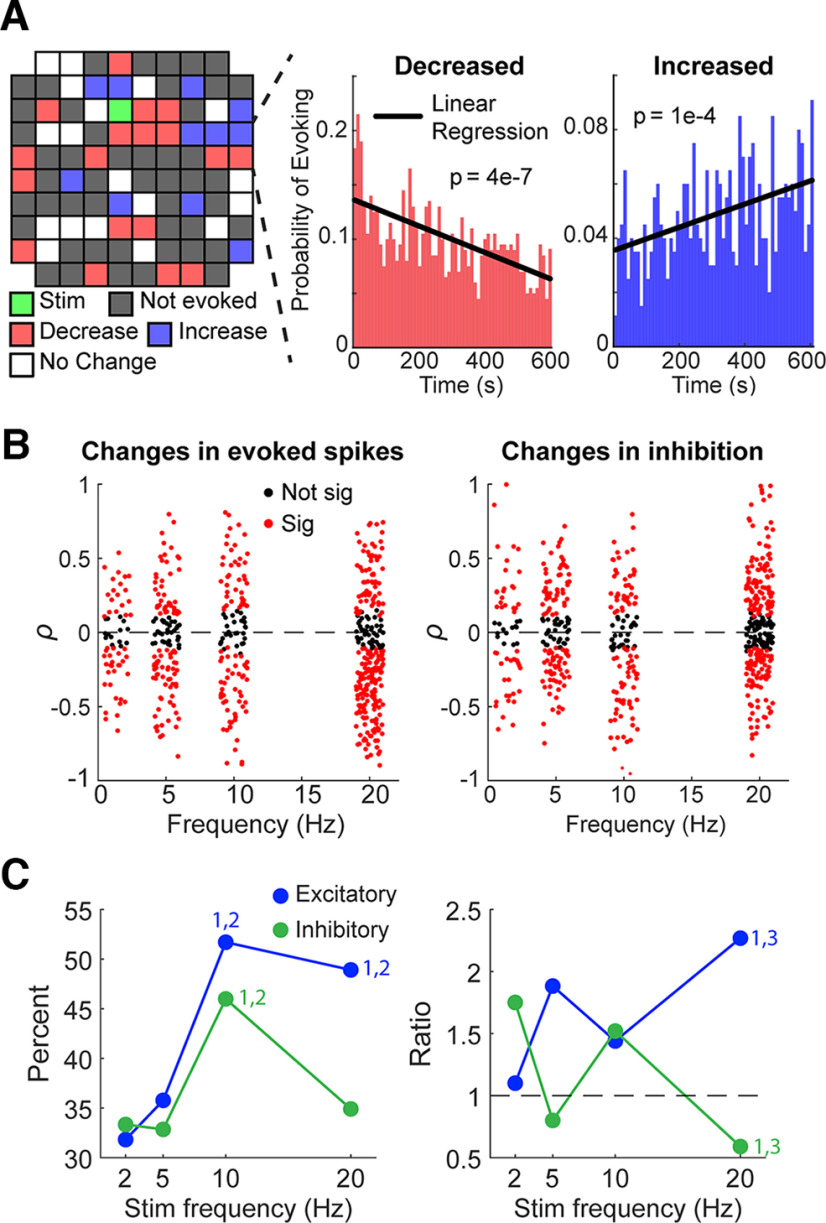
Changes in evoked spike probability and inhibition duration with repetitive stimulation. ***A***, Left, Changes in evoked spikes across the array during a session with 10-Hz repetitive stimulation. A random unit was chosen for each electrode to demonstrate the lack of spatial organization of changes in responses. Right, Examples of changes in the probability of evoking spikes increasing or decreasing over time. ***B***, Pearson correlation coefficients of evoked spike probability and inhibition duration plotted against stimulation frequency (*n* = 585). Note that experiments delivered tonic stimulation at 2, 5, 10, or 20 Hz; a jitter was added to the frequencies of each point to better visualize the data. ***C***, The percentage of spikes that had significant (*p* < 0.05) changes over time for each stimulation frequency for both evoked spike probability and inhibition (left) as well as the ratio of decreases to increases (right). The dashed line of the right plot shows a threshold, if the value is higher (>1) the changes induced are more likely to be decreasing whereas if the value is lower (<1) the changes are more likely to be increasing. Numbers above points denote significance (1: significant from 2 Hz, 2: significant from 5 Hz, 3: significant from 10 Hz; *p* < 0.05, ANOVA).

Repetitive microsimulation has been shown to modulate the responses of units to ICMS (Dadarlat et al., 2019; Michelson et al., 2019; Zucker and Regehr, 2002). We documented the effects of repetitive ICMS over the stimulus period ranging from 5 to 20 min on the evoked spike probability and the duration of inhibition by delivering stimuli at 2, 5, 10, or 20 Hz. The probability of evoking a spike often increased or decreased over time, following a linear or exponential trend over the course of the session (Fig. 9*A*).

The evoked spike probability was significantly more likely to change with higher frequencies of stimulation (Fig. 9*B*, left, *C*, left). Of the changes, high frequencies (20 Hz) were likely to cause decreases in evoke spike probability compared with lower frequencies. Changes in inhibition duration over time were also more likely to occur at higher frequencies, and high frequencies were more likely to cause increases in inhibition duration (Fig. 9*B*, right, *C*, right).

**Figure 10.**
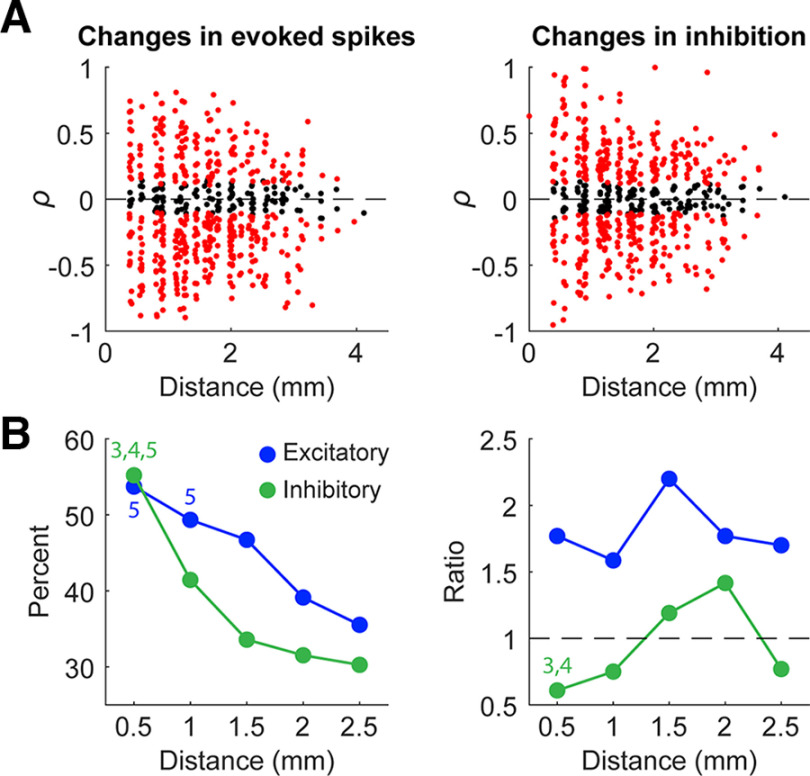
Changes with repetitive stimulation with respect to distance. ***A***, Pearson correlation coefficients of evoked spike probability and inhibition duration plotted against distance of the recorded unit from the stimulated site (*n* = 585). Note that experiments delivered tonic stimulation at 2, 5, 10, or 20 Hz; a jitter was added to the frequencies of each point to better visualize the data. ***B***, The percentage of spikes that had significant (*p* < 0.05) changes over time for each bin of distance (±0.25 mm around each point) for both evoked spike probability and inhibition (left) as well as the ratio of decreases to increases (right). We did not include data points from channels >2.75 mm from the stimulated site to this figure because of the lack of samples. Numbers above points denote significance (3: significant from 1.5 mm, 4: significant from 2 mm, 5: significant from 2.5 mm; *p* < 0.05, ANOVA).

The changes also depended on the distance from the recording site to the stimulated site (Fig. 10*A*). The probability of evoking spikes and duration of inhibition were significantly more likely to change the closer the units were to the stimulated site (Fig. 10*B*, left). The specific change in evoked spike probability did not have a dependence on distance, but inhibition duration was more likely to decrease in sites further from the stimulated site (Fig. 10*B*, right).

**Figure 11.**
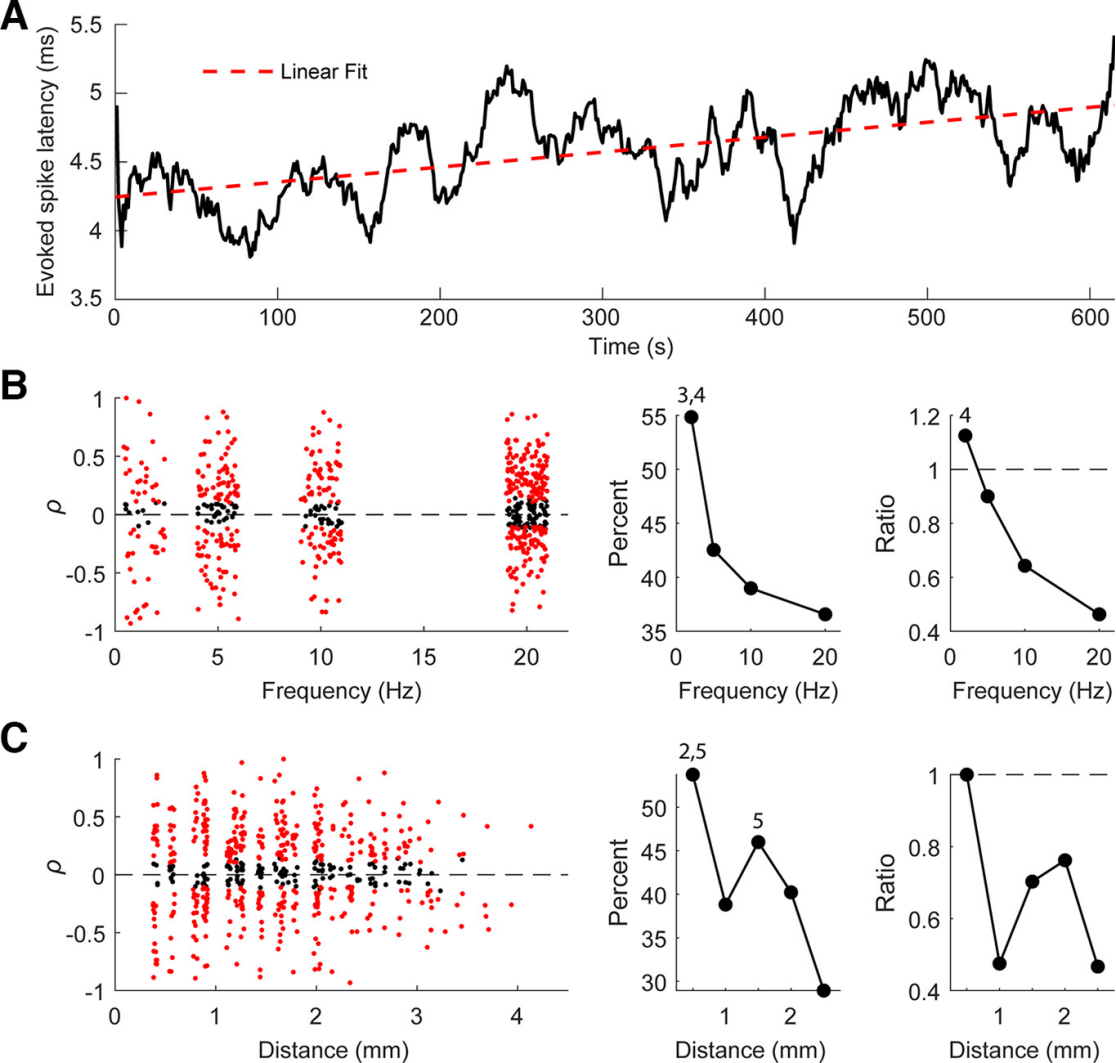
Changes in evoked spike latency with repetitive stimulation. ***A***, An example of evoked spike latency changing over time. ***B***, Scatter plot of Pearson correlation of the evoked spike latency over time with respect to stimulation frequency (left; *n* = 585). Percentage of evoked spike latencies with a significant change over time (middle), and the ratio of decreases to increases (right). Numbers above points denote significance (3: significant from 10 Hz, 4: significant from 20 Hz; *p* < 0.05, ANOVA). ***C***, Scatter plot of Pearson correlation of the evoked spike latency over time with respect to distance from the stimulated site (left; *n* = 585). Percentage of evoked spike latencies with a significant change over time (middle), and the ratio of decreases to increases (right). Numbers above points denote significance (2: significant from 1 mm, 5: significant from 2.5 mm; *p* < 0.05, ANOVA).

Finally, in 72/585 (12%) of units, we also observed changes over time in the latency of the evoked spikes (Fig. 11*A*). Latency changes typically occurred in units that were recorded on electrodes closer to the stimulation site, more commonly occurred with low frequency stimulation, and was more likely to increase over time (Fig. 11*B*). Of the units with changes in their evoked spike latency, 249/447 (56%) units had increasing latencies and 198/447 (44%) had decreasing latencies. Distance from the stimulated site also played a role, with units closer to stimulation more likely to have changes in their evoked spike latency over time (Fig. 11*C*).

### Relationship between evoked spikes and inhibition

**Figure 12.**
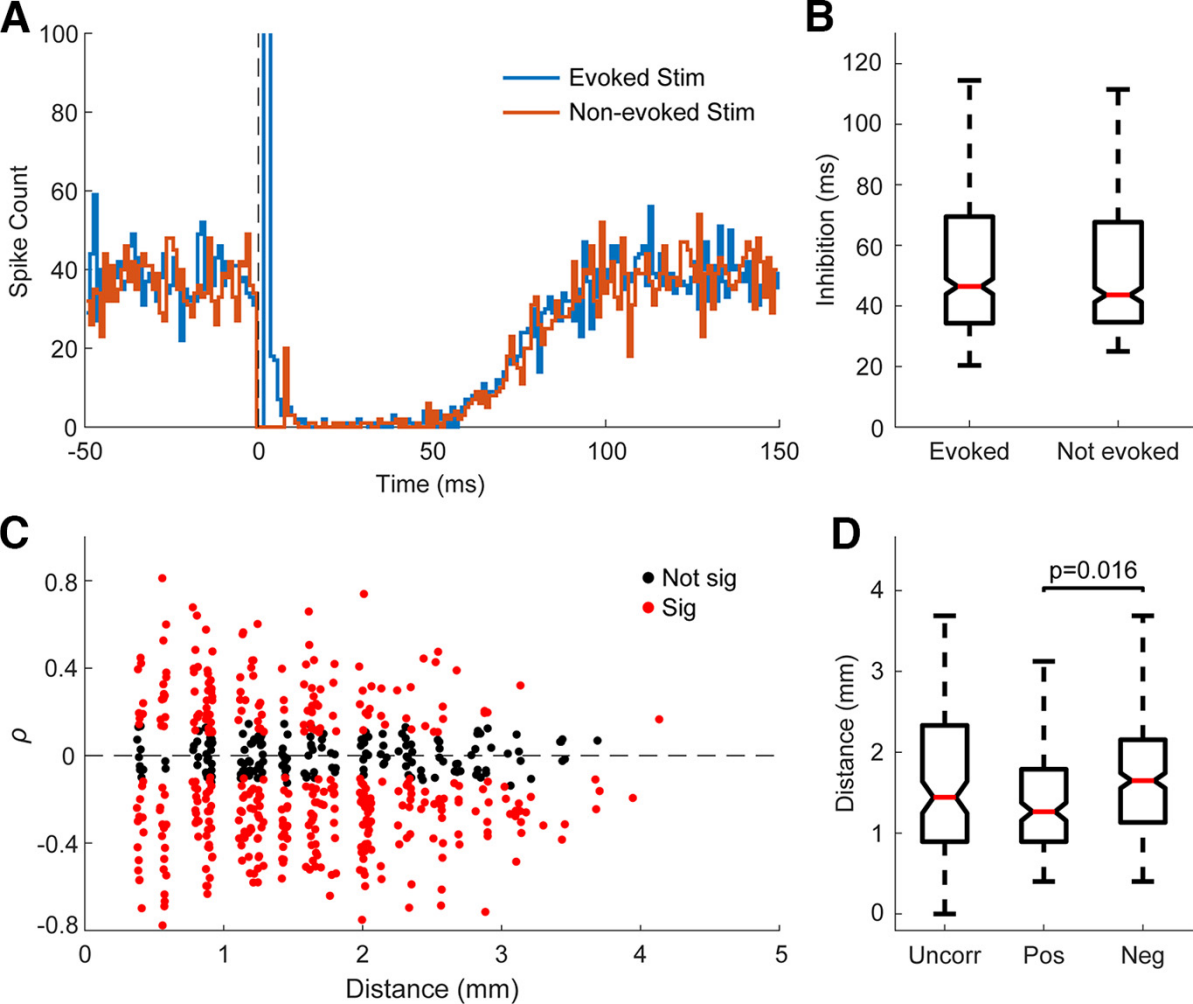
Relationship between evoked spikes and inhibition. ***A***, Example PSTH with 1-ms bins following stimuli that evoked spikes and those that did not demonstrating similar inhibitory response. Each condition consisted of 1500 stimuli. Smoothed (2 ms wide Gaussian moving window) PSTH of the two different stimulation classifications. Note the inhibition is extremely similar for both. ***B***, A comparison of the inhibition strength in the two different classifications for 470 units. There was no statistically significant pairwise difference between the two groups. ***C***, Scatter plot of the Pearson correlation coefficient between evoked spike probability and inhibition duration against distance of the recorded unit from the stimulated site (*n* = 585). ***D***, Comparisons of distance from the stimulus site of units with uncorrelated, positively correlated, and negatively correlated evoked spike probability and inhibition duration. The labeled *p*-value is from the Wilcoxon rank-sum test.

Since evoked spikes and inhibition often both exhibited changes over time, we sought to determine whether they were directly related on a trial-by-trial basis. Individual stimuli in each experiment were divided into two categories: those that evoked spikes and those that did not. For the unit in Figure 12*A*, the PSTHs of the two classifications of stimuli are very similar except for the evoked spike peak. We found no statistical pairwise difference between the inhibition induced by stimuli that evoked spikes compared with the stimuli that did not evoke spikes (585 units; Fig. 12*B*).

Although whether a stimulus evokes a spike does not affect the inhibitory response, the mechanisms underlying the changes over time could nevertheless be related. We analyzed the correlation between the probability of evoking a spike and duration of inhibition over time to determine whether they were positively or negatively correlated. Correlations with *p* < 0.05 were considered significant, and all other instances were denoted to be uncorrelated. Of the 585 units tested, 30% (173 units) had positively correlated changes in the probability of evoking spikes and the duration of inhibition, 47% (273 units) had negatively correlated changes, and 23% (135 units) were uncorrelated. Units with positively correlated evoked spike probabilities and inhibition duration tended to be closer to the stimulated site (Fig. 12*C*,*D*).

For each unit, there were unique, non-zero transmission delays between the onset of stimulation and when the evoked excitatory signal arrived at the unit to evoke it. Since each unit had a different average evoked spike latency, adding the transmission delays to the stimulation delays served as a means of normalizing the effect. Therefore, when we calculated the probability of evoking a spike as a function of the delay between the previous spike time and stimulation onset, we modified the delays by adding the average evoked spike latency of the unit to each of the delays.

### Cell type does not correlate with stimulus response properties

To determine whether the cell type of the recorded units influenced their responses to ICMS, we used the spike width, calculated as the time between the minimum value of the waveform to the maximum value, to classify each unit as fast-spiking (FS) or regular-spiking (RS; McCormick et al., 1985; Connors and Gutnick, 1990).

**Figure 13.**
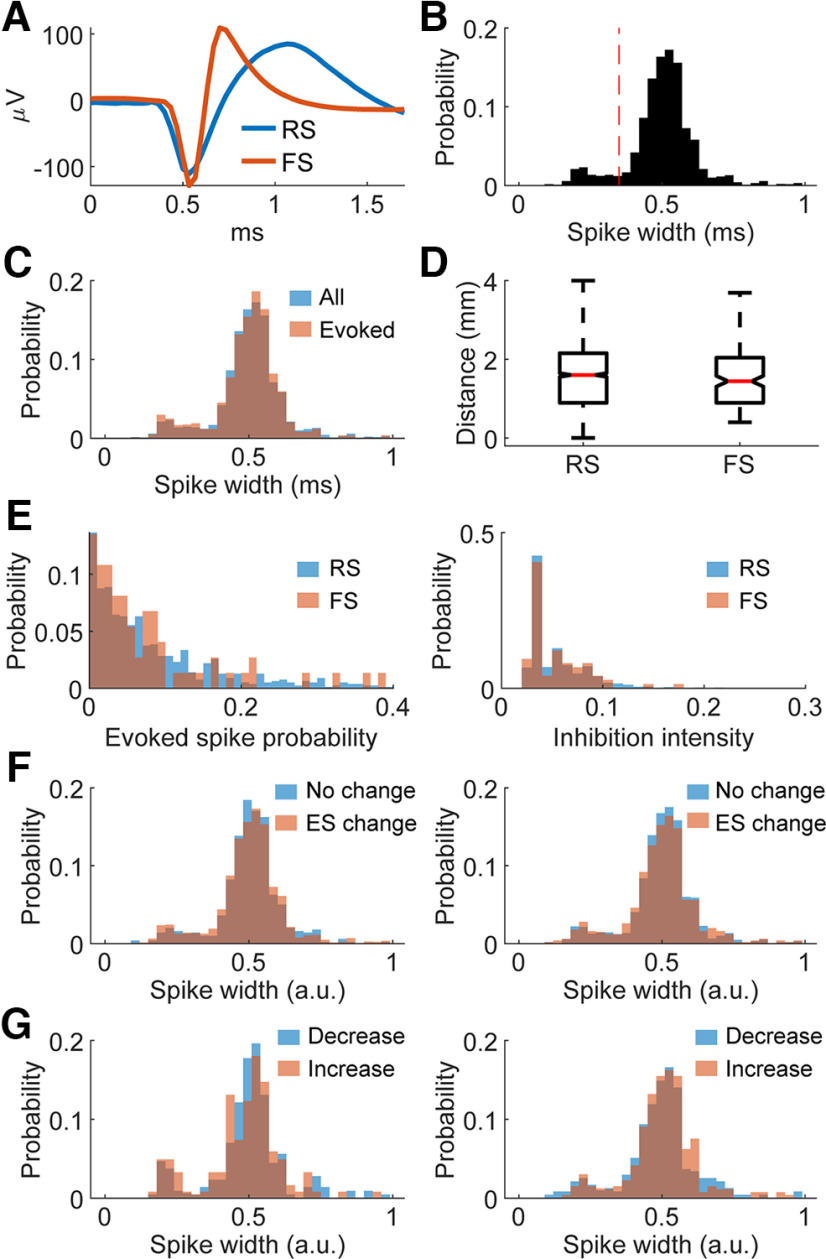
Comparisons between regular spiking and fast spiking neurons. ***A***, Example of regular spiking (RS) and fast spiking (FS) neuron waveforms. ***B***, Distribution of the widths of spike waveforms (trough to peak time). Vertical dotted line indicates the classification boundary (0.35 ms) – fast spiking neurons fall to the left and regular spiking to the right. ***C***, Spike width distribution of all recorded spikes and spikes that were evoked by stimuli. ***D***, Distance from the stimulated site of evoked spikes grouped by spike width. ***E***, Evoked spike probability distribution (left) and inhibition duration distribution (right) of RS and FS neurons. ***F***, Spike width distributions of evoked spike probability change versus no change over time (left) and inhibition duration change versus no change over time (right). ***G***, Spike width distributions of evoked spike probability decrease versus increase over time (left) and inhibition duration decrease versus increase over time (right).

Figures 13*A* shows an example of the two different spike waveforms, and Figure 13*B* shows the distribution of their spike widths. We separated the units into two groups based on their defined spike width shown at the dotted line in Figure 13*B*. Around 10% of all units fell to the left of the line and were denoted to be FS; the rest were denoted to be RS. We found that the putative cell type did not correlate with the distribution of distances from the stimulated site, whether a spike could be evoked, the probability of evoking a spike, the duration of inhibition, or how any measure changed over time (Fig. 13*C–G*). Thus, all results reported herein are independent of the type of unit recorded.

Although there are now increasingly complex methods of categorizing different cell types with extracellular recordings using various features of the units (Lee et al., 2021; Petersen et al., 2021), extracting firing characteristics of our units was compromised by the presence of stimulation. As such, this analysis is provided as a simple first step in assessing cell type; a more extensive analysis would be necessary to establish any differences with more confidence.

## Discussion

### Comparisons to previous studies

ICMS has been shown to predominantly activate neurons trans-synaptically (Gustafsson and Jankowska, 1976; Butovas and Schwarz, 2003; Hussin et al., 2015; Klink et al., 2017). We observed in our study that spike latencies fluctuated more than would occur with antidromic activation, and stimuli that were delivered within 1 ms after a spike were still able to evoke spikes. Since antidromic activation would result in collision and an absence of a spike at such short latencies, this suggests that the recorded units were predominantly activated orthodromically. Also consistent with previous experiments, we saw evoked spikes in units recorded up to 4.5 mm away from the stimulation site, which suggests that ICMS activates a distributed population rather than only a concentrated sphere of neurons around the electrode tip (Stoney et al., 1968; Butovas and Schwarz, 2003; Histed et al., 2009; Hao et al., 2016).

**Figure 14.**
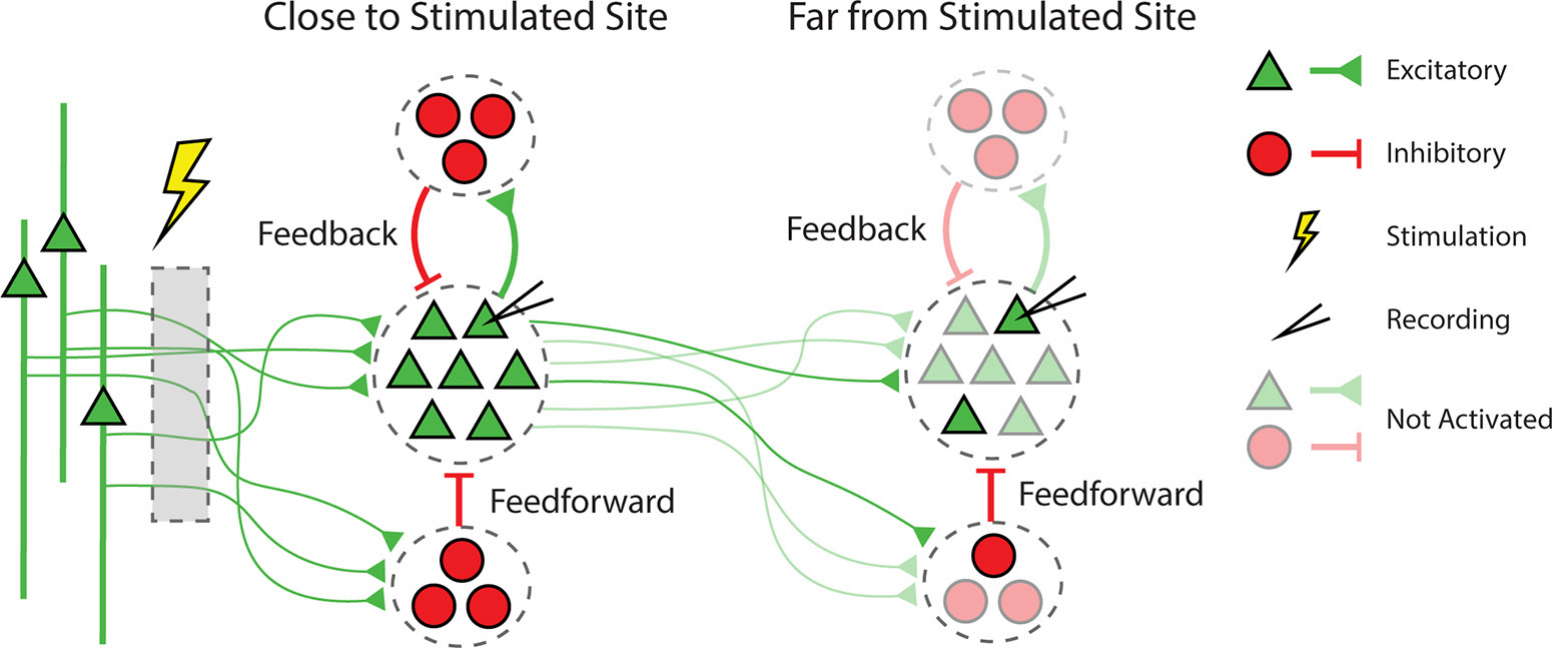
Stimulation response schematic. Schematic of cortical circuitry that generates excitatory and inhibitory ICMS responses through feedforward and feedback mechanisms. Stimulation activates axons projecting to the recording site. Sites closer to the stimulated site have more complete activation compared with sites further from the stimulated site. Possible direct connections from the stimulated site to the far site would also be sparsely activated.

The excitatory response was directly measurable in our experiments, but the subsequent inhibitory response manifested as a lack of spikes. Butovas and colleagues concluded that a similar inhibitory response to ICMS was likely caused by GABA_B_ receptors, which they confirmed with a follow-up study with pharmacological blocks (Butovas and Schwarz, 2003; Butovas et al., 2006). GABAergic inhibition would also be consistent with the similarity in the sigmoidal curves of evoked spikes and inhibition with stimulus amplitude in our experiments, if more excitatory neurons are excited by the higher intensity stimulation, more inhibitory neurons will be activated via feedforward and feedback circuits, thereby increasing the amount of inhibition (Fig. 14).

We found that inhibition typically lasted between 5 and 100 ms and rarely over 100 ms, which is significantly shorter than the average time constants of GABA_B_ inhibitory postsynaptic potentials of 150–200 ms (Connors et al., 1988; Bettler et al., 2004). Therefore, GABA_A_-mediated inhibition is a more likely candidate to explain our results. Previous studies have shown that GABA_A_ is involved in recurrent polysynaptic inhibition, which we were likely activating via ICMS (Silberberg and Markram, 2007; Zhu et al., 2011). The different animal models and recorded cortical region in these studies may account for these discrepancies.

ICMS activates long horizontal fibers to both feedforward inhibitory networks and the recorded excitatory neurons (Fig. 14). These fibers have stronger excitatory connections to the inhibitory interneurons than the principal cells, particularly in layer 2/3, which may explain why we sometimes observe an inhibitory response without any excitatory response (Matsumura et al., 1996; Cruikshank et al., 2007; Helmstaedter et al., 2008; Adesnik and Scanziani, 2010). This, coupled with the fact that inhibitory neurons often target somatic or perisomatic compartments (Isaacson and Scanziani, 2011; Rudy et al., 2011; Tremblay et al., 2016), suggests that the observed inhibition may be initially activated via feedforward circuitry, and is subsequently followed by the excitatory response. The excited principal cells may then activate feedback circuitry that contributes to the inhibitory response (Fig. 14).

### Stimulus response depends on network activity and intrinsic membrane properties

Previous research has shown that stimulus responses depend on network activity both *in vitro* (Weihberger et al., 2013; Kumar et al., 2016) and *in vivo* (Kara et al., 2002). Our findings were consistent with these results; the probability of evoking a spike was often positively correlated with spontaneous firing rate, whereas the duration of inhibition was often negatively correlated with firing rate.

English and colleagues demonstrated that the transmission probability for postsynaptic spikes of inhibitory neurons in the hippocampus *in vivo* is a function of the timing between the previous postsynaptic spike and presynaptic spike (English et al., 2017). Moreover, that study found that this dependency was independent of whether the previous postsynaptic spike was spontaneous or evoked, which suggested that intrinsic properties of the postsynaptic membrane were responsible for the dependence. Our results confirm and extend these findings by demonstrating that the timing of the previous spike affects not only the transmission probability for spontaneous presynaptic spikes, but also the stimulus-evoked spikes in cerebral cortical neurons. Furthermore, we found that the probability distributions for evoking a spike as a function of the timing between the previous spike time and stimulation onset and the spike train autocorrelation were often significantly positively correlated, which further reinforces the notion that this dependency reflects the intrinsic properties of the recorded units.

Altogether, our results reveal that the stimulus response has at least two dependencies other than stimulus amplitude and distance from stimulus site: the intrinsic membrane properties of the recorded neurons and the activity of the network. However, a sufficiently large stimulus current may saturate the responses and overcome these dependencies.

### Repetitive stimulation modulates stimulus responses

Michelson and colleagues showed that the number of neurons activated by electrical stimulation diminished over time with higher frequencies measured with calcium imaging in a 407 × 407 μm window, which was attributed to a diminishing region of activation (Michelson et al., 2019). Their study showed that changes began at regions distal from the stimulated site when delivering stimuli with frequencies >10 Hz. These results are consistent with our study across the larger spatial field (4 × 4 mm) of the Utah array, as evoked spikes were more likely to be diminished with higher frequency of stimulation at distances closer to the stimulated site. A large difference we observed was the time course of the changes; Michelson et al., reported the changes occurred within seconds and plateaued whereas we observed changes occurring for up to 20 min. Additionally, we also observed that in some units the probability of evoking a spike increased over time even at longer distances and higher frequencies, which cannot be fully explained by a diminishing region of activation.

Although we did not explicitly measure the duration of changes induced by repetitive stimulation, we observed that they typically lasted <2 min. Because of the short-lived nature of the induced changes, various mechanisms of short-term synaptic plasticity such as vesicle depletion and facilitation by calcium influx (Zucker and Regehr, 2002; Citri and Malenka, 2008) may best explain our results. Evidence suggests that different forms of short-term plasticity exist for synaptic connections between different cell types (Losonczy et al., 2002; Blackman et al., 2013). Beyond these differences, previous *in vitro* work by Markram showed that synaptic connections between pyramidal neurons of the same morphologic class and interneurons had similar facilitating and depressing characteristics, but with different time courses (Markram et al., 1998).

Together, these results may explain why the frequency-dependent changes that we measured were different for each unit. We did not discern any differences between regular and fast spiking neurons for any measure, but there are limitations in such cell type classifications with extracellular recordings. Furthermore, we also observed changes in the latency of evoked spikes because of repetitive stimulation, which has previously been shown to occur in the presence of short-term plasticity (Boudkkazi et al., 2007). The latencies typically changed more often with higher frequency stimulation in spikes closer to the stimulated site, similar to the evoked spike probability and inhibitory response changes. Future studies with specific differentiation between cell and synapse types may shed more light on whether cell type-specific differences account for the variability across spikes.

### Excitation and inhibition are independently activated but modulated together within an interconnected network

The balance between excitation and inhibition within the cortex is a much-studied topic and is highly relevant to neural computation. Although the examined network size, location, and synaptic connections vary greatly, the strong consensus is that excitation and inhibition are generally comodulated (Chen, 2004; Haider, 2006; Isaacson and Scanziani, 2011; Turrigiano, 2012; Xue et al., 2014; Rubin et al., 2017). Whether a stimulus evoked a spike on a trial-by-trial basis did not affect the subsequent inhibitory response, but we found that the probability of evoking a spike and the duration of inhibition were frequently positively or negatively correlated over time. Units close to the stimulated site typically had positively correlated evoked spike probability and inhibition duration, both of which were negatively correlated with firing rate. Units far from the stimulation channel, however, had positively correlated evoked spike probabilities and firing rates, which were both negatively correlated with inhibition duration.

The effect of distance can be explained by the fact that sites closer to stimulation are more likely to be activated by ICMS (Butovas and Schwarz, 2003; Hao et al., 2016). Because of the feedforward and feedback inhibitory circuitry, if the total excitation increased or decreased because of short-term plasticity, the inhibition should change in a positively correlated manner. Sites far from stimulation, however, are not activated as comprehensively and are thus less likely to be susceptible to short-term plasticity. Similarly, the negative correlations in evoked spike probability and inhibition at these far sites are likely because of network dynamics, whereas the positive correlations in closer sites are likely due changes in short-term plasticity caused by direct activation via ICMS.

In conclusion, ICSM activates excitatory horizontal fibers projecting to both excitatory and inhibitory circuitry. To ensure ICMS is as effective as possible, stimulation paradigms, especially those employed for prolonged periods of time, need to consider the interactions between excitation and inhibition, stimulation timing, as well as the possible induction of short-term plasticity leading to changes in responses over time. Maintaining low stimulation frequency and stimulation amplitude should provide the most consistent responses to stimulation, removing the confounding factors of short-term plasticity as well as artificial circuit interactions driven by strong activation of local networks. Further investigation into cell type-specific responses using optogenetics or pharmacological blocks will provide additional insights on single neuron responses to ICMS as well as the relevant cortical circuitry.

## Synthesis

Reviewing Editor: Nathalie Ginovart, Universite de Geneve Decisions are customarily a result of the Reviewing Editor and the peer reviewers coming together and discussing their recommendations until a consensus is reached. When revisions are invited, a fact-based synthesis statement explaining their decision and outlining what is needed to prepare a revision will be listed below. The following reviewer(s) agreed to reveal their identity: NONE.

The authors have adequately responded to the reviewer and the article can be accepted for publication provided that a few final minor corrections are made, as indicated in the following comments from the reviewer:

Reviewer:

Overall there authors were very responsive, and I am happy to say I am in favor of publication with only the text revisions mentioned below.

#1

In regards to the initial comment pertaining to original line 231 “if they are stimulus evoked how are they cortically activated? you mean timed in part by cortex?”

The reviewers answer is sufficient, but it would be helpful for a portion of readers to add a brief mention of this process.

#2

In regard to my original last comment involving the division of cell types in extracellular recordings, I accept that “complex” (meaning accurate but time consuming) cell separation may be beyond the scope of this study, and accept their current classification of cell types as sufficient.

#3 this ad hominem swipe at the endeavor of separating cell types in extracellular recordings is not necessary: “In

addition, studies have shown that extracellular recordings are often insufficient in properly

categorizing different cell types due to overlap in features collected by extracellular recordings

such as spike width and firing characteristics (Insel & Barnes, 2015; Lemon et al., 2021;

Vigneswaran et al., 2011). “

I would be fine with the text with that sentence removed, or at least qualified to say that this is more of a problem with certain specified recording devices/methods than others. Lee et al 2021 demonstrate progress in this realm in primates https://elifesciences.org/articles/67490

## Author Response

Reviewer 1 :

Very interesting paper with a few key suggestions for improvement.

The discussion in the manuscript regarding Figure 11 does not accurately reflect the data.

Specifically, it appears that latency changes occurred more commonly with low frequency stimulation.

This was a typo - it has been fixed to “low” to accurately reflect the figure (line 326).

The Figure 6 caption needs revision. Some aspects of the figure are not defined (e.g., “Not sig” and “Sig”).

Definition of the labels have been added to the figure caption.

It is indicated in the Figure 7 caption that, “An example of a neuron with positively correlated firing rate (black) and evoked spike probability (blue) over 10 minutes.” However, the right side of Figure 7A shows negative correlation. Additionally, the Figure 7 caption has 2 “C.” instances.

The caption has been fixed to reflect the figure.

There are additional typographical errors that need to be addressed: Page 15: add space in “4from” Page 16: move period in “. (Figure 9B right, 9C right)”

The typographical errors have been fixed (lines 294 and 318 respectively)

Reviewer 2 :

The biggest problem with the paper is that the statistics are nowhere near robust enough. Whole sections describe raw #s of neurons doing one thing and another raw # another, across animals, and make conclusions from this without any statistics at all. There are often p values with no associated n or even a test. The plots in the figures do not always match the sentence describing them and their statistical analysis. It is unclear how key metrics such as spike train correlations are performed, and their exclusion of certain spikes from specific analysis is not explained in enough detail to understand if it is kosher.

Conceptually this paper is very interesting, made even more appealing by the earnest and thorough approach. Lots of figures are great in this case. While I was very excited when I started reading, I quickly realized that there were analytical and statistical issues that made it impossible to judge the conclusions. There were often no statists reported, eg listing raw #s of cells that do one thing or another and drawing a conclusion with no test, and if so many times only p values were reported with no test or n given. There was just not enough information on the the choices of statistical tests, whether they were based on a priori power analysis, if it was checked if the 2 data were normally distributed (especially for very close to significant p values for tests that assume normality). The list is too long to go through them all, but basically every figure needs to have the statistics thoroughly re-evaluated, and in many cases should be cross validated (ie line 250), and distributions of data should be shown (ie line 242).

In the revised paper we have ensured that p-values and number of samples for statistical tests have been listed properly when presenting the results of each analysis.

We mentioned that we chose certain tests due to nonparametric (non-normal) nature of the data as well as due to making categorical comparisons in the Statistical Analysis section of the Methods (line 211). We have updated the section to better reflect our reasoning: “Two-sided Wilcoxon signed-rank test (signrank, MATLAB) was used to compare between groups due to the nonparametric nature of the data. Two-sided Wilcoxon rank-sum (ranksum, MATLAB) tests were used for paired data. Fisher’s exact test (fishertest, MATLAB) or two-way analysis of variance (ANOVA) (anova2, MATLAB) was used to compare pairs of or multiple groups of categorical data, respectively. The Pearson correlation was used for all correlation tests.

The Kolmogorov-Smirnov test was used as a non-parametric test to compare probability distributions. P-values for significance and tests used are reported in individual analyses.”

We repeated the shuffling process 100 times for both the pair and population level to validate the results and ensure it was not due to the instance of random shuffling (line 267).

We cannot show all distributions as it would result in hundreds of figures. Line 242 in the original document (currently line 268) discusses comparing pairs of spikes for each experimental session resulting in over 10000 pairs, which would not be feasible to show. As such, for the distributions that were unfeasible to demonstrate in full we have provided examples instead (mainly Figures 6B and 6C). All other analyses should demonstrate the full distribution of each measurement. more: line 140

This is questionable and needs to be demonstrated somehow. what do you mean higher temporal precision, after you just said you dont have enough data? you are saying effectively you know you cant get a good estimate, and are claiming a poor one is better?

We are calculating the inhibition duration by observing the timing of the next spontaneous spike after each stimulus. This provides us with higher temporal resolution because we can document inhibition duration per stimulus rather than having to collect 10’s or 100’s of stimuli to generate a PSTH. We have adjusted the text to more clearly explain our approach and reasoning. Starting on line 142: “While evoked spike timing can easily be determined with the PSTH due to their high probability and narrow time window, the duration of the inhibitory response occurs over a 3 broader window of time. Therefore, a large number of stimuli is required to get a densely sampled PSTH to measure inhibition strength, which prevents measuring changes in inhibitory strength with fine temporal resolution. Thus, rather than using the PSTH, we measured the duration of inhibition on a stimulus-by-stimulus basis by calculating the time between the onset of each stimulus and the first non-evoked spontaneous spike” line 150 to clarify then the first evoked AND first spike after stim, their ISI to the last spike is disregarded? We are not entirely certain what this is asking. The line that is referenced states that if there wasn’t a spike between two consecutive stimuli, we considered the first stimulus to not have a measurable inhibition duration (designated as NaN. The doublet- and triplet-stimuli experiments demonstrate that the inhibition duration is “reset” during each stimulus. These instances were rare and mainly occurred in units with very low firing rates. We have updated the text to clarify our approach on line 151: “If a second stimulus occurred prior to a spontaneous spike, the duration of inhibition was measured with respect to the second stimulus rather than the first, as we found that each stimulus “reset” the period of inhibition (Figure 3).” line 168 why?

We measured how the timing between the previous spike time and stimulation onset determined the evoked spike probability. If two consecutive stimuli occurred without a spike in between, the first stimulus is a confounding factor in measuring the distribution. If we kept these stimuli, rather than measuring how the timing of the previous spike relative to the stimulation onset influenced the probability distribution, we may have instead measured how the timing between the two stimulation pulses influenced it. As a result, we removed these stimuli when considering this measure. line 170 need to validate this somehow or show the raw data too

We have updated the text to explain our reasoning on line 181: “For each unit, there were unique, non-zero transmission delays between the onset of stimulation and when the evoked excitatory signal arrived at the unit to evoke it. Since each unit had a different average evoked spike latency, adding the transmission delays to the stimulation delays served as a means of normalizing the effect. Therefore, when we calculated the probability of evoking a spike as a function of the delay between the previous spike time and stimulation onset, 4 we modified the delays by adding the average evoked spike latency of the unit to each of the delays.” line 189 how did you come up with 3%? A low number of data points makes it difficult to document correlation analyses for changes over time. If we want a significance level of 0.05, power of 0.8, and expect the correlation to be roughly 0.3, we require 85 samples. The experiments with the fewest stimuli had roughly 3000 stimuli. 3% of 3000 is 90, which is the number of samples we need to perform the analyses with enough power assuming each data point occurs at a different time bin. line 231

if they are stimulus evoked how are they cortically activated? you mean timed in part by cortex?

We believe the evoked spikes are trans-synaptically activated, i.e. the stimulation excites a group of fibers near the electrode tip which projects onto the neurons we are recording from. Thus, we are not necessarily directly activating the recorded neurons but indirectly through the cortical fibers (thus “cortically activated”). There is a wealth of literature that supports this mechanism referenced in the manuscript (for example, Histed et al., 2009; Logothetis et al., 2010; Butovas & Schwarz 2003; Gustafson & Jankowska 1976). line 237 need to clarify this logic

If two units have a high probability of being evoked, there is a higher probability that they will be evoked by the same stimulus. In an extreme example, if two units were evoked 100% of the time, we would observe that their evoked activity was 100% correlated. In less extreme cases, where the units had lower, but still high evoked spike probabilities, it would be difficult to discern whether large correlations in evoked activity reflected them both independently having high evoked spike probabilities, or whether they were part of an underlying network that excited them together. We have tweaked the text to clarify this reasoning in line 256: “Though there exist statistically significant correlations (2904 out of 12419 tested pairs, or ∼23%), this may be a consequence of the units within the pairs independently having high probabilities of being evoked, rather than being correlated due to an underlying network state, thereby artificially inflating their correlation strength.” 5 line 242 why is 8% meaningless? We did not mean to imply that 8% is meaningless, just that it is rather low. We have updated the text to remove any confusion (line 268): “We found that on average 1013 ({plus minus}8.3 standard deviation) out of the 12419 tested pairs (∼8.2% {plus minus}0.06% standard deviation) had significantly different distributions (Fisher’s exact test, p<0.05), but a large majority did not.” line 250 you need to describe how you are shuffling and why. The previous paragraph explains the process of shuffling and the reasoning why (line 256). In short, we need to decouple the possibility of high probabilities of being evoked affecting the correlation analysis. As a result, we shuffled which stimuli evoked which spikes to create a baseline comparison to compare distributions as a way to compensate for the different evoked probabilities.

Last thing, need to better separate cell types than just spike width, eg firing rate, ACG properties including bursting, etc. Check out CellExplorer

Different cell types are difficult to distinguish with only extracellular recordings with heavily overlapping and sometimes even conflicting features. We are aware of more extensive categorization methods using various features of extracellular recordings, but it is difficult to extract these features in the presence of stimulation, and we believed this to be outside the scope of the experiment. We did not wish to suggest our categorization was comprehensive and just wanted to provide a simple check to see if there are any obvious differences. The text has been updated to reflect this reasoning on line 368: “Although there are now increasingly complex methods of categorizing different cell types with extracellular recordings using various features of the units (Lee et al., 2021; Petersen et al., 2021), extracting firing characteristics of our units was compromised by the presence of stimulation. In addition, studies have shown that extracellular recordings are often insufficient in properly categorizing different cell types due to overlap in features collected by extracellular recordings such as spike width and firing characteristics (Insel & Barnes, 2015; Lemon et al., 2021; Vigneswaran et al., 2011). As such, this analysis is provided as a simple first step in assessing cell type; a more extensive analysis would be necessary to establish any differences with more confidence.”

I would be happy to re-review this paper with robust analysis and statistics
